# SERS-Based Assessment of MRD in Acute Promyelocytic Leukemia?

**DOI:** 10.3389/fonc.2020.01024

**Published:** 2020-06-29

**Authors:** Cristina Turcas, Vlad Moisoiu, Andrei Stefancu, Ancuta Jurj, Stefania D. Iancu, Patric Teodorescu, Sergiu Pasca, Anca Bojan, Adrian Trifa, Sabina Iluta, Alina-Andreea Zimta, Bobe Petrushev, Mihnea Zdrenghea, Horia Bumbea, Daniel Coriu, Delia Dima, Nicolae Leopold, Ciprian Tomuleasa

**Affiliations:** ^1^Department of Hematology, Iuliu Hatieganu University of Medicine and Pharmacy, Cluj-Napoca, Romania; ^2^Department of Hematology, “Ion Chiricuta” Institute of Oncology, Cluj-Napoca, Romania; ^3^Faculty of Physics, Babeş Bolyai University, Cluj-Napoca, Romania; ^4^Research Center for Functional Genomics and Translational Medicine, Iuliu Hatieganu University of Medicine and Pharmacy, Cluj-Napoca, Romania; ^5^Medfuture Research Center for Advanced Medicine, Iuliu Hatieganu University of Medicine and Pharmacy, Cluj-Napoca, Romania; ^6^Department of Hematology, Carol Davila University of Medicine and Pharmacy, Bucharest, Romania

**Keywords:** DNA methylation, acute promyelocytic leukemia, measurable residual disease, disease monitoring, patient follow-up

## Abstract

Acute promyelocytic leukemia (APL) is characterized by a unique chromosome translocation t(15;17)(q24;q21), which leads to the PML/RARA gene fusion formation. However, it is acknowledged that this rearrangement alone is not able to induce the whole leukemic phenotype. In addition, epigenetic processes, such as DNA methylation, may play a crucial role in leukemia pathogenesis. DNA methylation, catalyzed by DNA methyltransferases (DNMTs), involves the covalent transfer of a methyl group (-CH3) to the fifth carbon of the cytosine ring in the CpG dinucleotide and results in the formation of 5-methylcytosine (5-mC). The aberrant gene promoter methylation can be an alternative mechanism of tumor suppressor gene inactivation. Understanding cancer epigenetics and its pivotal role in oncogenesis, can offer us not only attractive targets for epigenetic treatment but can also provide powerful tools in monitoring the disease and estimating the prognosis. Several genes of interest, such as RARA, RARB, p15, p16, have been studied in APL and their methylation status was correlated with potential diagnostic and prognostic significance. In the present manuscript we comprehensively examine the current knowledge regarding DNA methylation in APL pathogenesis. We also discuss the perspectives of using the DNA methylation patterns as reliable biomarkers for measurable residual disease (MRD) monitoring and as a predictor of relapse. This work also highlights the possibility of detecting aberrant methylation profiles of circulating tumor DNA (ctDNA) through liquid biopsies, using the conventional methods, such as methylation-specific polymerase chain reaction (MS-PCR), sequencing methods, but also revolutionary methods, such as surface-enhanced Raman spectroscopy (SERS).

## Background on DNA Methylation

Realizing that all cells in an organism are derived from a single cell (the zygote) and that they share an almost identical genetic information has left open a crucial question: what is the molecular substrate accounting for the differences between cell types. At the beginning of the twentieth century, Waddington has famously proposed that lineage commitment happens akin to a falling ball bound by tracts and walls consisting of epigenetic modification (1–4). Subsequent discoveries have clearly demonstrated that epigenetic changes indeed orchestrate ontogeny and that this molecular mechanism is perverted and hijacked in malignant diseases against the benefit of the organism itself (5). In this review, we describe the emerging epigenetic landscape of acute myeloid leukemias (AMLs). In particular, we focus on acute promyelocytic leukemia (APL) and the promising applications of DNA methylation in early disease detection, therapeutic decision, outcome prediction, measurable residual disease (MRD) monitoring.

Epigenetics refers to any change in the phenotype which does not imply an alteration in the DNA sequence (5). Among epigenetic modifications, DNA methylation was the first to be described (6). It consists in the covalent transfer of a methyl (-CH3) group from S-adenosyl-L-methionine (SAM) to the carbon 5 position of cytosine residue resulting in the modified base 5-methylcytosine (5-mC) (7). In the human genome, DNA methylation predominantly occurs at CpG dinucleotides (8). The symmetry of the CpG dinucleotides (which are mirrored as GpC in the opposite DNA strand) makes place for a methylation maintenance mechanism which can recognize newly formed hemi-methylated DNA and faithfully methylate the daughter DNA strand (7).

**Figure 1:**
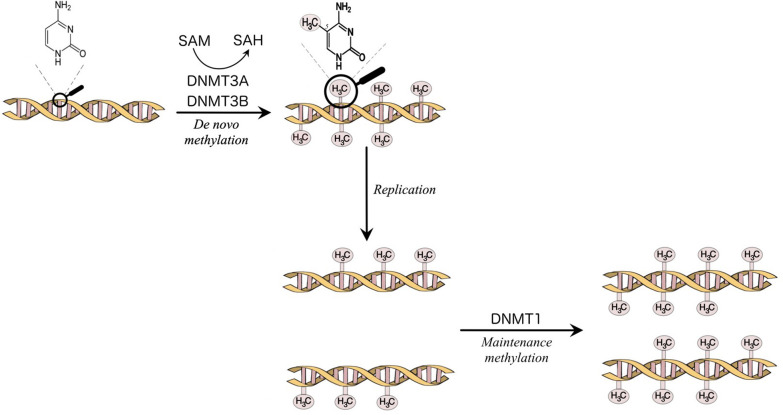
Methylation of the carbon 5 position of cytosine is catalyzed by DNA methyltransferases (DNMTs). S-Adenosyl methionine (SAM) acts as a methyl donor in the methylation reaction and is converted to S-Adenosylhomocysteine (SAH). DNMT3A and DNMT3B are responsible for the establishment of new DNA methylation patterns. DNMT1 mediates the maintenance of established patterns of methylation after replication (7).

The enzymes that catalyze DNA methylation are called DNA methyltransferases (DNMTs). The DNMTs are divided into *de novo* DNMT, DNMT3A, and DNMT3B, which create new methylation marks, and maintenance DNMT, DNMT1, which is responsible to maintain the established patterns of methylation when the cell divides [(7); Figure 1]. DNA methylation is a fundamental function in embryonic development, X-chromosome inactivation, genomic imprinting, preservation of genomic stability, transposable elements repression (5, 7, 9).

The opposing mechanism to DNA methylation, DNA demethylation, can take place actively, via the action of Ten-eleven translocation (TET) methyl cytosine dioxygenases, which progressively oxidize 5-mC to 5-hydroxymethylcytosine (5-hmC), 5-formylcytosine (5-fC), and 5-carboxylcytosine (5-caC) (10). In addition, passive demethylation can also take place by the gradual dilution of 5-mC during DNA replication, albeit only in the case of *de novo* methylation outside the setting of CpG islands. 5-mC can spontaneously deaminate to thymine (C → T transitions), such that 5-mC and consequently CpG dinucleotides have been progressively depleted during phylogenetic evolution and thus became under-represented in the human genome (11, 12). The Krebs cycle carboxylic acid 2-oxoglutarate acts as a cofactor for TET2, while succinate inhibits TET2 (13). We will see bellow how perturbations in these two carboxylic acids can bring about similar DNA hypermethylation phenotypes, acting as cancer metabolites.

A CpG cluster is called a CpG island and is identified in certain regulatory regions of the genome, including the promoter regions of genes. Such islands are usually un-methylated and active from a transcriptional point of view (11, 14). When a cell becomes malignant, it locks genes (e.g., tumor suppressor genes) in the “off” position by gene promoter methylation and thus transcription is suppressed (11, 14). Using this mechanism, decreased global DNA methylation, together with a selective hypermethylation of promoter gene regions, may be hallmarks of cancer and may differentiate malignant from normal DNA (15). DNA methylation may alter the expression of a gene by direct or indirect mechanisms (7). Thus, DNA methylation interferes directly with the transcriptional machinery of a cell by diminishing the affinity of transcription factors for promoters. However, a recent survey of more than 500 transcription factors found decreased affinity for methylated promoters in only 20% (16). Indirectly, DNA methylation inhibits transcription via DNMTs, which recruit H3K9 histone methyltransferases and histone deacetylases as well as via 5-mC readers possessing methyl-CpG-binding domains (MBDs) (17). This is how a repressive chromatin structure is produced. This close cooperation between DNA methylation and various epigenetic changes establishes the solid maintenance of gene expression changes during cell division and differentiation (7).

As methylation is important in cellular physiology, it is only natural that it also plays key roles in various conditions varying from inflammatory disorders and cardiovascular diseases to cancer (18–22). Indeed, somatic DNMT3A mutations are found in ~15–35% of cases of acute myeloid leukemias, the vast majority being loss of function missense mutations in arginine 882 (R882) (23). R882 mutations reduce the methylation activity of the enzyme by some 80%, acting in a dominant negative fashion on the formation of the DNMT3A tetramers. As expected, R882 leads to focal but genome-wide DNA hypomethylation that drives malignant progression via genetic de-repression (24). Less expected was the finding that loss of function TET2 mutations, which results in a hypermethylated DNA, also inflicts a growth advantage to hematopoietic stem cells and drive leukemogenesis (25). How come that haploinsufficiency in two enzymes with opposing functions can converge on the same malignant phenotype? The answer seems to lie in the reduction of 5-hmC content that underlies the loss of function of either DNMT3A or TET2. On one hand, 5-hmC is the end-product of the reaction catalyzed by TET2, such that TET2 deficiency directly results in decreased 5-hmC DNA content (26). On the other hand, it is expected that DNMT3A deficiency depletes 5-mC levels, thus translating in diminished substrate availability for TET2. The co-occurrence of DNMT3A and TET2 mutations in a significant proportion of hematological malignancies adds another layer of complexity to the problem, pointing toward their non-redundant effect on cellular proliferation. Indeed, double knockout mice (Dnmt3a^−/−^, Tet2^−/−^) exhibit accelerated leukemogenesis compared to both Dnmt3a^−/−^ and Tet2^−/−^ mice (27). Methylome analyses of double knockout mice demonstrated regions of independent, competitive, and cooperative activity underscoring a complex repression of lineage-specific transcription programs (27).

The mutual exclusion between isocitrate dehydrogenase (IDH) mutations and TET mutations in AML was the first clue hinting at the downstream convergence of these seemingly disparate molecular pathways (28). Subsequent studies demonstrated that the following mechanism takes place: neomorphic IDH mutations confers a novel function to the enzyme, which switches from α-ketoglutarate formation to 2-hydroxyglutarate (2-HG) production. The latter acts as a competitive inhibitor of 2-oxoglutarate for TET2 (29). The result is the same as with a *bona fide* loss of function mutation in TET2.

The molecular axis outlined above (IDH → 2-HG → TET2/DNMT → hyper/hypo methylation → decreased 5-hmC) was the setting for several new classes of drugs, among which IDH inhibitors are the last to enter the scene (29). Several clinical trials highlighted a marked response in patients with IDH-mutant AML that underwent therapy with IDH-inhibitors. For instance, the response rate to ivosidenib, an IDH-1 inhibitor, in patients with relapsed or refractory IDH-mutant AML was over 40% (30). A remarkably interesting observation was that patients undergoing therapy with IDH inhibitors exhibited a differentiation syndrome akin to the differentiation syndrome seen in APL patients (31). The similarities between IDH-mutant AML and APL has been recently substantiated by Boutzen et al. who reported that 2-HG reprograms the molecular machinery of the cells for responding to retinoids (32). The results suggested that IDH mutation results in the upregulation of CCAAT/enhancer binding protein α (CEBPα), which is a key regulatory switch enough for induction of differentiation toward the granulocyte lineage (32, 33).

## APL Pathogenesis

APL, a unique biological and clinical variant of AML, accounts for up to 10–15% of newly diagnosed acute myeloid leukemias in adults (34). APL typically presents with coagulation abnormalities which eventually lead to life-threatening disseminated intravascular coagulation and diffuse bleeding (34). The therapeutic advancements over the last decades dramatically improved the prognosis and decreased the mortality of the disease (34, 35). The cytogenetic hallmark of APL is a balanced reciprocal chromosomal translocation t(15;17) which involves the juxtaposition of the promyelocytic leukemia (PML) gene on chromosome 15 and the retinoic acid receptor alpha (RARA) gene on chromosome 17. This results in the chimeric PML/RARA gene formation (14, 36, 37). PML/RARA is the driving oncogene in APL (38) and is responsible for the maturation arrest at the promyelocytic stage (34). This differentiation blockage leads to the absence of mature myeloid cells and overproduction of promyelocytic blast cells, which accumulate in the bone marrow and blood (34, 36, 39).

In myeloid cells, RARA forms heterodimers with retinoid X receptor (RXR) and binds to specific DNA sequences called retinoic acid-responsive elements (RAREs), which are found in regulatory regions of target genes (40, 41). Physiologically, retinoic acid (RA) binds to these heterodimers and activates target genes transcription, including genes that are important in myeloid differentiation (39–42). Thus, RARA plays a key role in the differentiation of hematopoietic stem cells in humans by acting as a transcription factor for several downstream genes involved in differentiation (43).

When the ligand (RA) is not present, RARA/RXR heterodimers are able to recruit corepressors, such as nuclear receptor co-repressor (N-CoR), and inactivate transcription (14, 34, 44). Physiological concentrations of RA determine the corepressors to dissociate and the heterodimers to recruit coactivators, thus restoring transcription (14, 44).

PML/RARA has reduced sensitivity to physiological concentrations of RA and forms stable complexes with histone deacetylases (HDAC), DNMTs and Polycomb group proteins to target promoters of RARE-containing genes (14, 45). These complexes disrupt the transcriptional programs described above. Therefore, PML/RARA expression abrogates the retinoic acid dependent myeloid differentiation (14, 34, 46). In contrast, pharmacological doses of all-trans retinoic acid (ATRA) result in the dissociation of corepressor molecules, promoting transcriptional derepression, and terminal differentiation of promyelocytic blast cells into neutrophils (14, 39).

Although being considered the central effector of APL, PML/RARA cooperates with additional epigenetic changes to induce the whole leukemic phenotype, changes that will be further detailed below (34, 36).

## Methylation in Acute Promyelocytic Leukemia

The product of the PML gene is a protein that is the key organizer of the PML nuclear bodies (PML-NBs) (47). PML-NBs are found in the nuclear matrix of cells and consist of multiple proteins, all recruited by PML (47, 48). The functions of these NBs range from tumor suppression, angiogenesis, differentiation, senescence to response to viral infections, stress, and DNA damage (48). In APL cells, on the other hand, PML/RARA fusion transcript disrupts the integrity and function of PML nuclear bodies and delocalizes PML from nuclear bodies to hundreds of micro-speckles (48, 49). Cells with PML-NBs dysfunction show reduced ability to undergo senescence and apoptosis (48). Moreover, the pattern of DNMT1 and DNMT3A is diffuse in normal cells, whereas in cells expressing PML/RARA, the methyltransferases are delocalized to the newly formed micro-speckles. This implies that the DNMTs nuclear compartmentalization is modified by PML/RARA recruitment (49, 50).

Promoters that contain a CpG island but no RAREs are not modified by the expression of PML/RARA, which suggests that PML/RARA binds to specific genes that contain RAREs and subsequently induces transcriptional silencing (49). Retinoic acid receptor beta (RARB), a gene with a RARE, is one of these specific genes, involved in the differentiation blockage in the PML/RARA transfected cells (49). Upon treatment with either 5-Azacytidine or HDAC inhibitors, transcription of RARB2 was only partially released, whereas simultaneous treatment with the two agents led to complete transcription of the target gene. This indicates that two mechanisms are involved in the process of transcriptional suppression induced by PML/RARA: recruitment of HDAC (by RAR moiety) and recruitment of methyltransferases (by PML moiety) (49).

**Figure 2:**
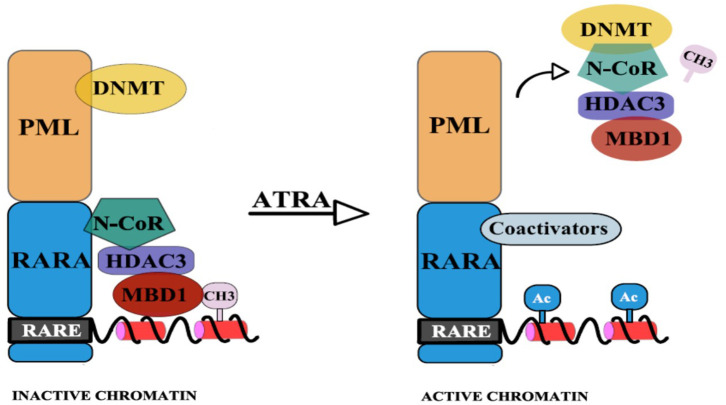
PML-RARA, through its PML moiety establishes interactions with DNMTs. Newly methylated CpGs are used as “docking sites” for MBD1 which can also recruit other corepressors. The RAR moiety interacts with the nuclear corepressor N-CoR, which further recruits HDAC3. HDAC3 can also interact with the TRD of MBD1. This results in hypoacetylation, DNA methylation, and subsequent gene silencing. Upon ATRA treatment, the stable complex is disassembled and coactivators (with histone acetyltransferase activity) are recruited (14).

To reinforce this hypothesis, Villa et al. have emphasized the interdependence of the two epigenetic pathways: DNA methylation and histone deacetylation (14). They showed that the physical binding of PML/RARA at the RARB2 locus, co-occurs with DNMT3A recruitment and hypermethylation of RARB2 promoter (14). The CpGs that become newly methylated are next used as “docking sites” for Methyl-CpG-binding domain protein 1 (MBD1), which in association with an HDAC3-mediated specific mechanism determine silencing of gene expression. Thus, a corepressor complex that involves MBD1, DNMT3A, and HDAC3 is formed (14). Pharmacological doses of RA lead to the disassembly of the stable complex, releasing MBD1 and HDAC3 from PML/RARA, thus promoting partial transcriptional derepression [(14); Figure 2]. Moreover, MBD1 also has a direct and independent impact on gene silencing. This is indicated by the fact that mutations in the MBD and in the trans-repression domain (TRD) of MBD1 can restore transcription and prevent the differentiation blockage at the promyelocytic stage. Also, MBD1, in association with suboptimal concentration of PML/RARA, can restore hypermethylation of RARB2 (14).

Furthermore, besides having an important structural role in the repression complex, HDAC3 can also directly and independently impact gene expression (14). Consistent with this, by using interference RNA (RNAi) to reduce the expression of endogenous HDAC3 in NB4 cells, the endogenous RARB2 mRNA is induced (14).

RARA gene has two major isoforms: RARA1 and RARA2 (40). The promoter P2 that generates mRNA for RARA2 contains a RARE, whereas the promoter P1 generating mRNA for RARA1 does not. This suggests that RARA2 is an RA-inducible protein having a critical role in granulopoiesis and myeloid differentiation (40). RARA P2 methylation is specifically involved in the initiation and pathogenesis of APL, occurring with comparable frequencies in a cohort of 63 APL patients studied at diagnosis and at relapse (11). Aberrant P2 methylation's role in leukemogenesis was also confirmed in the NB4 cell line, where the un-translocated RARA was P2 methylated. Consistent with this, the RARA transcript could not be detected by RT-PCR. After 5-Azacytidine treatment, P2 demethylation, parallel with RARA2 re-expression were observed. But despite these molecular changes induced by 5-azacytidine, in the absence of RA there was no evidence of differentiation. Thus, only pharmacological doses of ATRA can overcome the differentiation block related to RARA epigenetic changes (11).

In addition to these findings, in APL, RARA was shown to be dysregulated by both translocations, forming the PML/RARA fusion transcript, and epigenetics where the untranslocated RARA is P2 methylated (11). However, P2 methylation did not affect prognosis (11).

According to Chim et al. epigenetic dysregulation of tumor suppressor genes contributes to APL pathogenesis (51). They determined the frequency of p15 and p16 gene promoter methylation from serial marrow DNA from 26 APL patients. Using methylation-specific polymerase chain reaction (MS-PCR), they indicated that p15 methylation is frequent in APL (73% of cases). P16 methylation, on the other hand, is not frequently found, suggesting that it might not play a part in initiating the disease, but it can be acquired during clonal progression (51). Persistent p15 methylation preceded hematological relapse. Correlated with inferior disease-free survival (DFS), p15 methylation is a valuable marker for MRD monitoring and prognosis prediction. Thus, p15 methylation in APL is correlated with a poor prognosis in a multivariate analysis for age, sex, and initial leukocyte count (51). The same group reported a second study on 29 APL patients and analyzed the clinicopathologic and prognostic impact of the following genes: p15, p16, RARB, estrogen receptor (ER), E cadherin (E-CAD), p73, caspase 8 (CASP8), VHL, MGMT (52). The data showed abnormal methylation of five of the nine genes: p15, ER, RARB, p16, E-CAD, without methylation of the other genes. The high number of aberrantly methylated genes found in APL indicates that they may be critical in leukemogenesis (52). However, among them, the only significant marker affecting survival and prognosis remained p15 methylation (52). Methylation of this tumor suppression gene was associated with an inferior disease free survival (DFS) (*p* = 0.008), but did not impact overall survival (OS) (*p* = 0.88) and thus became the single negative prognostic variable in a multivariate analysis, along with several other variables (leucocyte count at diagnosis, age, sex) (*p* = 0.019) (52).

As it is frequently associated with hematological malignancies, the p15 cyclin-dependent kinase inhibitor, has been extensively studied in APL (51–54). Teofili et al. have conducted a study on a series of 65 APL cases treated according to the AIDA protocol (53, 55). They identified three categories of patients: patients with either complete, or partial, or without p15 methylation. Patients with complete methylation of p15 had a higher rate of relapse and a lower DFS when compared to patients with partial or without p15 methylation. OS, however, was not influenced in any of the three groups (53). Fifty-two percent of cases had p15 hypermethylation, this being the largest APL cohort investigated for p15 methylation (53). As previously stated, in Hong Kong, Chim et al. reported hypermethylation in 73% of a 26-patient cohort (51). The difference is probably linked to the small number of patients, as well as to the high relapse rate reported for the Asian cohort, with a 5-year DFS being smaller than expected (51). Moreover, Chim et al. in their studies did not examine if the aberrant p15 gene methylation resulted in the absence of p15 expression (51, 52). This link is of utmost importance, as evidenced below.

The gene p15 is not expressed in the presence of fully methylated DNA but expressed in patients with partially methylated DNA (53). Also, p15 promoter methylation *per se* is not the key criterion for identifying patients at increased relapse risk. Aberrant methylation is associated with a negative prognosis only if it culminates in the total loss of p15 expression (53). Baba et al. in 37 patients with APL showed p15 methylation in 16 cases (43.2%) (54). Out of these 16 cases, seven had partial methylation and nine were found to have complete methylation. Surprisingly, nine patients in this study had leukocytosis at diagnosis and were found to have a complete p15 methylation. Thus, they noted an important association between leukocyte count at diagnosis, regarded as a negative prognostic factor, and the pattern of p15 methylation (54). During maintenance therapy, six patients relapsed, all with complete methylation of p15, with no p15 mRNA detected by RT-PCR (54). Interestingly, in this study, p15 methylation significantly decreased DFS, but in line with prior studies, it did also not influence OS (54). A complete methylation and loss of p15 gene expression may end up causing increased risk of relapsing. This adds to the growing body of evidence that hypermethylation of p15 promoter has a pivotal in APL leukemogenesis and can be considered a reliable biomarker for prognosis estimation and follow-up in APL (54).

In APL, blasts have higher DNA methylation levels and increased variability compared to normal CD34 positive cells, promyelocytic cells, and remission bone marrow cells (38). However, Schoofs et al. contrary to prior studies, had a different answer to the question of whether aberrant DNA methylation is critical in APL initiation or if aberrant methylation of DNA is a late event in the pathogenesis of the disease (38). Using reduced representation bisulphite sequencing (RRBS), they report no differences in the global DNA methylation pattern of cells of pre-leukemic PML/RARA knock-in mice and their healthy counterparts, thus demonstrating limited impact of DNA methylation on APL initiation (38). Moreover, retroviral transduction of PML/RARA into Lin- bone marrow cells caused the typical differentiation block, without important differences in DNA methylation. Also, ATRA treatment led to successful differentiation without evidence of short-term changes in the DNA methylation pattern (38). Thus, opposed to prior reports, they showed that DNA methylation modifications are involved in leukemogenesis, but rather as a second-hit, and independent of PML/RARA binding. PML/RARA is the cardinal oncogene in APL and aberrant DNA methylation patterns are associated with leukemia phenotype but constitute later events in the progression of the disease contributing to its maintenance rather than to its initiation (38).

Phenotypic features, such as increased age, FLT3-ITD mutations, high Sanz score or early death are associated with more hypermethylated than hypomethylated differentially methylated regions (DMRs) (38). Various transcription factor–binding sites, as is the case of c-myc–binding sites are found to have decreased methylation (38). On the other hand, REST- and SUZ12-binding sites from human embryonic stem cells (hESC) were identified in regions of preferentially high methylation in APL (38). Whereas, in the past some papers showed that PML/RARA acts by recruiting DNMTs to its target gene promoters (56), the genome-wide DNA methylation analysis of Schoofs et al. identified only three PML/RARA binding sites that were hypermethylated, including the previously reported RARB2 promoter (38). DNA methylation was not identified at PML/RARA targets such as RARA, DNMT3A, and RUNX1, their results suggesting that PML/RARA might in fact prevent methylation of target sites and protect genes against epigenetic inactivation (38). In line with these statements, other works reported that transcription factor binding protects from methylation (57). This model implies that loss of protective transcription factor binding and not direct DNMT recruitment is responsible for the abnormal pattern of DNA methylation (38).

A large fraction of DNA methylation is also observed in gene bodies and is implicated in gene activation rather than transcriptional suppression (38). The positive correlation between gene-body methylation and gene expression is in contradiction to the classic theory of increased methylation of gene promoters in cancer, suggesting that in APL, DNA methylation is not associated only with gene promoters (38).

Abnormal DNA methylation is present throughout all chromosome regions, but preferentially noticeable at chromosomal ends (38). Interestingly, hypomethylation is the main epigenetic change detected at chromosomal ends, these hypomethylated regions being particularly observed at the ends of four chromosomes, namely, 5, 7, 9, and 17 (38).

Subramanyam et al. have published one of the first works that tested the cooperativity *in vivo* between an oncogene and epigenetic changes (36). They showed that higher DNMT3A1 expression collaborates with PML/RARA and leads to the development of APL with shortened latency, greater penetrance, and earlier lethality on transplantation into irradiated recipients compared with cells from PML/RARA mice (36). The possible cooperation between PML/RARA and DNMT3A in the initiation of APL is suggested by the fact that mutations in DNMT3A are almost never found with t(15;17) that creates the PML/RARA onco-fusion gene (58). Experiments on DNMT3A-null mouse showed that, in a retroviral transduction system, expression of PML/RARA was not able to promote aberrant self-renewal when DNMT3A was absent (58). The presence of a fully functional DNMT3A was also necessary for *ex vivo* aberrant self-renewal promoted by PML/RARA, as neither DNMT3B, nor the two mutant DNMT3A genes that are deficient in catalytic activity (DNMT3A R882H and DNMT3A Q615^*^) were able to re-establish myeloid self-renewal (58).

This data supports that functional methyltransferase activity of DNMT3A is mandatory for both *in vivo* and *ex vivo* PML/RARA-induced phenotypes (58).

Whereas, Cole et al. reported that absent or unfunctional DNMT3A protects Ctsg-PML/RARA mice against APL (58), Mayle et al. and Yang et al. argued that after a long period of latency, DNMT3A deficient mice are prone to developing both myeloid and lymphoid malignancies (59, 60).

## Effects of Treatment on APL's Epigenetic Landscape

**Figure 3:**
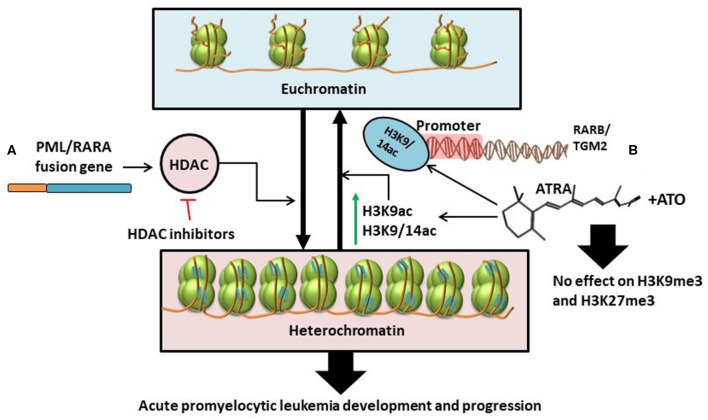
**(A)** PML/RARA fusion gene stimulates the activity of histone deacetylases thus causing the transformation of euchromatin (transcriptionally active) into heterochromatin (transcriptionally inactive). This results in the development and progression of APL. The effects of this chain of events could be disrupted with the help of HDAC inhibitors (61, 62). **(B)** ATRA as single treatment or in combination with ATO specifically increases the levels of H3K9ac and H3K9/14ac leading to chromatin acetylation. H3K9/14ac enrichment following treatment is particularly targeted at the promoter regions of RARB and TGM2. Combination treatment with ATRA and ATO has little to no effect on histone methylation (H3K9me3 and H3K27me3) (34).

In contrast to results from previous studies, technological advancements in genome-wide epigenetic studies revealed a limited role of DNA methylation in APL pathogenesis (61). Instead, PML/RARA induces a hypo-acetylated chromatin conformation by recruiting Histone deacetylases. Furthermore, ATRA induces significant increase in histone acetylation (H3K9ac, H3K9/14ac), without changes in histone methylation (H3K27me3 and H3K9me3), and without changes in DNA methylation at the binding sites of PML/RARA/RXR (61). All this data might suggest that histone deacetylation, and not histone or DNA methylation, is the key epigenetic mechanism involved in the major transcriptional repression role of PML/RARA and advocates for the advantages of HDAC inhibitors in the treatment of APL [(61, 62); Figure 3A].

With the expanding use of ATRA and arsenic trioxide (ATO) in the clinical setting, several works have been conducted to study the effects of the combination treatment on the epigenome and transcriptome (34). Single agent-treated cells revert to non-differentiated cells after treatment (34, 63–66). In contrast, combination therapy with ATRA and ATO induces a sustained state of terminal granulocytic differentiation, decreasing the relapse-rate (34, 67). To understand whether sustained epigenetic changes are responsible for the lasting effects of the combination therapy, Huynh et al. analyzed two cell lines, NB4 and NB4-MR2, the latter being resistant to ATRA. They investigated the effects of ATRA and ATO on cells during treatment vs. at 72- and 96-h following treatment interruption (34). The Canadian group suggests that persistent terminal granulocytic differentiation after combination treatment can be explained by the increased transcript levels of RARB, TGM2, CCL2, and ASB2, noticed in NB4 cells 96 h after treatment interruption (34). However, this hypothesis has not been validated in ATRA-resistant NB4-MR2 cells. In the ATRA-resistant cell line, although combination treatment resulted in more pronounced gene expression 72 h post-therapy, the transcript levels were not maintained after another 24 h, with even higher doses of ATO (34). To further assess the exact epigenetic mechanism that produced changes in gene expression, histone alteration was investigated by chromatin immunoprecipitation (ChIP), and CpG methylation by bisulphite-pyrosequencing. The ChIP-qPCR analysis showed that ATRA determines consistent enrichment of H3K9/14ac at the RARB and TGM2 promoters, that is not further increased with combined therapy of ATRA and ATO (34). This suggests that H3K9/14c is not the most important mechanism for the greater gene expression observed with combination treatment. Also, both ATRA alone and combination treatment have negligible impact on H3K9me3 and H3K27me3 marks associated with gene silencing [(34); Figure 3B].

Bisulphite pyrosequencing data reveals that ATRA reduces overall methylation, but to a lesser degree than combination treatment with ATRA and ATO (34). Combined therapy of the two agents reduces the aberrant methylation of CpG sites in the promoter regions of RARB and TGM2 in a dose dependent manner. Thus, the higher the dose of ATO, the greater the demethylation (34).

Long interspersed nucleotide element-1 (LINE-1) constitute repetitive DNA retrotransposons that comprise ~17% of the human genome (34). These are transposable elements, with the ability to move around the genome, to create insertional mutations and contribute to genomic instability. Being heavily methylated, the extent of LINE-1 methylation serves as a surrogate marker of global DNA methylation. NB4 and NB4-MR2 cells treated with ATRA or ATO or with combination treatment do not have changes in LINE-1 methylation levels, indicating that reduced CpG methylation in response to treatment is not genome-wide, but restricted to target genes (34).

When looking at mice transplanted with cells collected from the bone marrow of preleukemic APL transgenic mice, after treatment with 5-Azacytidine, the mice developed a marked acceleration of leukemogenesis (68). Altered expression of several genes is reported in the 5-Azacytidine-treated group. Such, they report the down-regulation of DMTF1 (a cyclin D–binding protein that has a growth-suppressive function), FOXO1A (a tumor suppressor gene), Bach2 (growth suppressor), DUSP1 (down-regulator of various MAPK signaling pathways and protein kinases involved in cell cycle control), and sestrin 1 (transcriptional target of the tumor suppressor p53). Scaglioni et al. also report up-regulation of Birc1e (IAP inhibitor that counteracts cell death), Sept9 (growth suppressor), and angiopoietin-like 4 (pro-metastatic gene). All this gene alteration implies that 5-Azacytidine, through its epigenetic modifications, can promote leukemogenesis (68), which is surprising given the fact that according to previous studies, PML/RARA-induced hypermethylation and gene silencing is an important mechanism in APL pathogenesis (68).

TP53 is among the most extensively studied tumor suppressor genes in cancer (69–71). Still, TP53 is very rarely mutated in APL (72–75). Ng et al. report that alternative epigenetic mechanisms have been proposed for the dysregulation of TP53 in APL (72). Thus, DAPK1 and p14ARF (both being involved in the positive regulation of p53) and microRNA (miR)-34a and miR-34b/c (p53's direct transcriptional targets) have been investigated for their promoter methylation (72). By using MS-PCR analysis, they report a complete methylation of miR-34a, miR-34b/c, and DAPK1 in the NB4 cell line, suggesting a tumor-specific role of their methylation in APL. Following 5-Azacytidine treatment, all investigated genes or microRNAs had a promoter demethylation and subsequent transcriptional activation in the NB4 cell line (72). miR-34b exerts its leukemogenicity function by upregulation of CREB and Cyclin A1. The miR-34b overexpression in NB4 transfected cells culminated in a significant decrease of cellular proliferation, which validates its tumor suppressive function in APL (72). The same group analyzed the samples from a cohort of 60 APL patients at diagnosis, and 43% had miR34-b/c methylation, but none were methylated for DAPK1, p14ARF, and miR-34a (72). However, miR-34b/c methylation did not influence OS or EFS, suggesting that further research in the field is compulsory (72).

## Surface-Enhanced Raman Scattering (SERS) as an Innovative, High-Sensitive Technique in Cancer Diagnosis, Prognosis, and Monitoring

Raman Spectroscopy is a non-destructive form of optical spectroscopy that relies on the interaction of light with the vibrational energy structure of chemical bonds of molecules and detects the inelastic scattering of laser photons. Subsequently, the frequency-shifted photons provide detailed information about the molecular structure of the sample (76, 77).

Nonetheless, the concentration of many metabolites is beneath Raman's detection limit (78). Thus, its low sensitivity often limits its use. To overcome this challenge and enhance the sensitivity of the method, Surface-enhanced Raman scattering (SERS), a technique that involves metal nanostructures for the amplification of the Raman signal of molecules, has been developed (76, 78). When the target of interest adsorbs onto nanometer-sized metal substrates such as silver or gold colloidal nanoparticles, the Raman signal of the target molecule is dramatically augmented (77, 79).

In the last decade, SERS has attracted much attention in the field of biomedical research. Several studies have investigated SERS as a method of cancer detection. Promising results have been shown in different types of malignancies (76–82). In AML, for example, the DNA methylation landscape translates into specific spectral differences. In accordance with the well-known global hypomethylation of malignant DNA, the SERS analysis of DNA from AML cells detected low intensity of the 1,005 cm^−1^ band, compared to normal DNA, the 1,005 cm^−1^ band corresponding to 5-methylcytosine. (76) Furthermore, the epigenetic modifications also influence the adsorption of DNA onto metal surfaces, culminating in increased SERS intensities for adenine in cancer DNA. The bands with higher SERS intensities that were attributed to adenine were bands 730 and 1,328 cm^−1^ (76).

These findings agree with the observations of Sina et al. which also showed that cancer-specific hypomethylation leads to higher affinities of cancer DNA to metal nanoparticles, thus enhancing its detection (80).

Moreover, by detecting the cancer-associated unique DNA methylation pattern, the SERS analysis of genomic DNA from circulating blood could discriminate between AML cases and healthy cases with increased sensitivity (82%), specificity (82%), and overall accuracy (82%) (76).

Daum et al. investigated the possibility of detecting the DNA methylation changes in living cells (81). By analyzing human colon carcinoma (HCT116) cells which are hippomorphic for DNMT1, and HCT116 wildtype cells, Raman microspectroscopy, and imaging could discriminate between low and high-methylation levels in both cell types. The results were confirmed by quantifying the global DNA methylation levels by anti-5mC immunofluorescence (IF) staining and 5mC enzyme-linked immunosorbent assay (ELISA). 1,257, 1,331, and 1,579 cm^−1^ Raman bands have been proposed as potential markers for monitoring DNA methylation (81).

Lin et al. employed SERS for quantifying small changes in DNA molecules at single nucleobase level (79). Their further attempt to detect blood circulating DNA achieved remarkable diagnostic sensitivity (83.3%) and specificity (82.5%) for discriminating patients with nasopharyngeal tumors and controls. (79) Their work demonstrated that SERS can be a rapid, low-cost, and sensitive liquid biopsy method used in the screening of nasopharyngeal cancer (79).

Encouraging results have also been obtained in studies aiming to demonstrate the possibility to use the SERS spectra of urine as a revolutionary screening tool for breast and prostate cancer. In both cases, the spectral differences could differentiate between breast cancer patients and controls and between prostate cancer patients and normal samples with remarkable accuracies (78, 82).

Good classification accuracies of SERS have also been demonstrated on a set of 253 serum samples obtained from patients diagnosed with oral, colorectal, ovarian, breast, lung cancer, and healthy controls (77). Interestingly, the SERS analysis did not just differentiate cancer patients from controls, but it could also assign cancer samples to their corresponding cancer types. Using principal component analysis–linear discriminant analysis (PCA-LDA), SERS correctly diagnosed the various cancer types with accuracies of 88, 86, 80, 76, and 59% for oral, colorectal, ovarian, breast, and lung cancer, respectively (77).

## DNA Methylation as a Surrogate Marker for MRD Detection

Aberrant patterns of DNA methylation are among the most frequently found epigenetic modifications in AML (83). However, these abnormal methylation patterns are not restricted only to AML as they occur in almost any hematologic or non-hematologic malignancy. Moreover, various studies provide evidence that DNA methylation plays a key role in APL pathogenesis and can be detected in clinical remission samples. Abnormal levels of methylation in remission are regarded as potent predictors of the relapse risk. This encourages researchers to believe that DNA methylation can be used as a widely applicable surrogate marker for the detection of measurable residual leukemia (83).

MRD techniques have undergone major technological advances ranging from identifying leukemia-associated immunophenotypes by multicolor flow-cytometry (MFC), to detecting gene mutations by RT-qPCR, digital droplet PCR (ddPCR), and next-generation sequencing (NGS) (84). However, choosing the technique that is best suited for personalized approaches still seems to be a challenging task. Recent years have opened the field of MRD to epigenetics, and in particular to methylation studies (85, 86). A variety of methodologies exist to assess methylation. These methodologies can be essentially divided into two classes: genome-wide studies and gene-specific studies (87).

Whole genome bisulphite sequencing (WGBS) is the “gold standard” of global DNA methylation studies (88). When DNA is treated with bisulphite the non-methylated cytosine deaminates and transforms into uracil. By PCR-amplification and consecutive sequencing analysis the converted residues will be replaced by their analog, thymine. 5-mC, on the other hand, is not modified by bisulphite treatment and will be read as cytosine. Therefore, the comparison of the sequencing reads from bisulphite treated and untreated DNA enables the identification of methylated cytosines (88, 89). The major advantage of WGBS is that it offers a comprehensive analysis of global methylation and identifies differentially methylated regions at single-nucleotide resolution (87, 88). However, its high cost, the difficult analysis of the NGS data and the bioinformatic expertise needed to assess its results are drawbacks that often limit its use (88). Moreover, by bisulphite treatment, the complexity of the genome is reduced to three bases (except for the 5-mC that is rather rare) and DNA comprising only three nucleotides can be hard to assemble (88). Also, the fragmentation of the DNA, which is part of the method, might lead to the formation of chimeric product genes after amplification (88).

MS-PCR, the prototype of target genes studies, allows the amplification of specific genes after bisulphite treatment, using two pairs of primers: one pair for the methylated DNA and the other pair for the unmethylated DNA (87, 88). Its obvious strengths are the ability to analyse candidate gene regions and the possibility to perform the method without expensive instrumentation (87). On the other hand, the primer design can introduce various biases and consequently lead to wrong amplification (87).

Nonetheless, none of the methods aiming at studying the methylation status has emerged as a reference standard technique (87). SERS, on the other hand, given the remarkable results mentioned in the research studies above, promises good differentiation of cancer from normal cells based on detecting spectral differences that emerge from the unique pattern of DNA methylation of cancerous cells (76). What makes SERS clinically applicable in the point-of-care setting is its low cost, high sensitivity, and specificity in detecting subtle changes in DNA methylation in samples collected by minimally invasive procedures (77, 79). Furthermore, compared to WGBS, SERS does not require DNA amplification, thus being easier to use and less time consuming (76, 79).

Given the fact that SERS has been widely investigated in the assessment of many malignancies, but especially in AML, it is only natural to imply that its use can be extrapolated to APL. APL is associated with a particular methylation pattern (38, 52), which could be reliably used for MRD monitoring with the help of SERS. Therefore, research needs to be fuelled in this field for warranting early diagnosis, outcome prediction, MRD monitoring and, all in all, personalized care for APL patients.

## Conclusion

The scientific research in epigenetic modulation has witnessed tremendous progress since its discovery. Among the epigenetic modifications, aberrant DNA methylation has recently emerged as one of the most frequent and well-described changes involved in hematological malignancies. In APL, secondary epigenetic events such as aberrant DNA methylation have been proposed to function in conjunction with PML/RARA. Therefore, understanding the important role of DNA methylation in APL pathogenesis may help in the development of novel strategies to use this biomarker in clinical settings for diagnosis and prognosis estimation. Furthermore, with the accurate detection capacity of SERS, the future of monitoring measurable residual disease promises exciting and impactful discoveries ahead.

## Author Contributions

All authors listed have made a substantial, direct and intellectual contribution to the work, and approved it for publication.

## Conflict of Interest

The authors declare that the research was conducted in the absence of any commercial or financial relationships that could be construed as a potential conflict of interest. The reviewer CS declared a past co-authorship with several of the authors SP, CTo, PT, DD, and VM to the handling editor.
